# Correction for Kennan et al., “Genomic Evidence for a Globally Distributed, Bimodal Population in the Ovine Footrot Pathogen Dichelobacter nodosus”

**DOI:** 10.1128/mBio.02560-19

**Published:** 2019-10-29

**Authors:** Ruth M. Kennan, Marianne Gilhuus, Sara Frosth, Torsten Seemann, Om P. Dhungyel, Richard J. Whittington, John D. Boyce, David R. Powell, Anna Aspán, Hannah J. Jørgensen, Dieter M. Bulach, Julian I. Rood

**Affiliations:** a Australian Research Council Centre of Excellence in Structural and Functional Microbial Genomics, Monash University, Victoria, Australia; b Department of Microbiology, Monash University, Victoria, Australia; c Norwegian Veterinary Institute, Oslo, Norway; d Department of Bacteriology, National Veterinary Institute, Uppsala, Sweden; e Department of Biomedical Sciences and Veterinary Public Health, Swedish University of Agricultural Sciences, Uppsala, Sweden; f Victorian Bioinformatics Consortium, Monash University, Victoria, Australia; g Life Sciences Computation Centre, Victorian Life Sciences Computation Initiative, Carlton, Victoria, Australia; h Farm Animal and Veterinary Public Health, Faculty of Veterinary Science, the University of Sydney, Camden, New South Wales, Australia

## AUTHOR CORRECTION

Volume 5, issue 5, e01821-14, 2014, https://doi.org/10.1128/mBio.01821-14.

It has been drawn to our attention that there are errors in the list of SRA accession numbers in Table S6 of this article, specifically in the last column of this table. Accordingly, we have provided a corrected copy of Table S6. The table has been replaced online. The authors apologize for any inconvenience that this error may have caused to other researchers.

